# Mild Gestational Diabetes and Adverse Pregnancy Outcome: A Systemic Review and Meta-Analysis

**DOI:** 10.3389/fmed.2021.699412

**Published:** 2021-07-05

**Authors:** Razieh Bidhendi Yarandi, Mojtaba Vaismoradi, Mohammad Hossein Panahi, Ingjerd Gåre Kymre, Samira Behboudi-Gandevani

**Affiliations:** ^1^Department of Biostatistics, University of Social Welfare and Rehabilitation Sciences, Tehran, Iran; ^2^Faculty of Nursing and Health Sciences, Nord University, Bodø, Norway; ^3^Department of Epidemiology, School of Public Health and Safety, Shahid Beheshti University of Medical Sciences, Tehran, Iran

**Keywords:** adverse maternal outcomes, adverse neonatal outcomes, mild gestational diabetes, meta-analysis, diagnostic criteria

## Abstract

**Background and Objectives:** Mild gestational diabetes (GDM) refers to the gestational hyperglycemia, which does not fulfill the diagnostic criteria for GDM. The results of studies on adverse pregnancy outcomes among women with mild GDM are controversial. Therefore, the aim of this systematic review and meta-analysis was to investigate the impact of mild GDM on the risk of adverse maternal and neonatal outcomes.

**Methods:** A thorough literature search was performed to retrieve articles that investigated adverse maternal and neonatal outcomes in women with mild GDM in comparison with non-GDM counterparts. All populations were classified to three groups based on their diagnostic criteria for mild GDM. Heterogeneous and non-heterogeneous results were analyzed using the fixed/random effects models. Publication bias was assessed using the Harbord test. DerSimonian and Laird, and inverse variance methods were used to calculate the pooled relative risk of events. Subgroup analysis was performed based on mild GDM diagnostic criteria. Quality and risk of bias assessment were performed using standard questionnaires.

**Results:** Seventeen studies involving 11,623 pregnant women with mild GDM and 53,057 non-GDM counterparts contributed to the meta-analysis. For adverse maternal outcomes, the results of meta-analysis showed that the women with mild GDM had a significantly higher risk of cesarean section (pooled RR: 1.3, 95% CI 1.2–1.5), pregnancy-induced hypertension (pooled RR: 1.4, 95% CI 1.1–1.7), preeclampsia (pooled RR: 1.3, 95% CI 1.1–1.5) and shoulder dystocia (pooled RR: 2.7, 95% CI 1.5–5.1) in comparison with the non-GDM population. For adverse neonatal outcomes, the pooled relative risk of macrosomia (pooled RR = 0.4, 95% CI: 1.1–1.7), large for gestational age (pooled RR = 1.7, 95% CI: 1.3–2.3), hypoglycemia (pooled RR = 1.6, 95% CI: 1.1–2.3), hyperbilirubinemia (pooled RR = 1.1, 95% CI: 1–1.3), 5 min Apgar <7 (pooled RR = 1.6, 95% CI: 1.1–2.4), admission to the neonatal intensive care unit (pooled RR = 1.5, 95% CI: 1.1–2.1), respiratory distress syndrome (pooled RR = 3.2, 95% CI: 1.8–5.5), and preterm birth (pooled RR = 1.4, 95% CI: 1.1–1.7) was significantly increased in the mild GDM women as compared with the non-GDM population. However, the adverse events of small for gestational age and neonatal death were not significantly different between the groups. Analysis of composite maternal and neonatal outcomes revealed that the risk of those adverse outcomes in the women with mild GDM in all classifications were significantly higher than the non-GDM population. Also, the meta-regression showed that the magnitude of those increased risks in both composite maternal and neonatal outcomes was similar.

**Conclusion:** The risks of sever adverse neonatal outcomes including small for gestational age and neonatal mortality are not increased with mild GDM. However, the increased risks of most adverse maternal and neonatal outcomes are observed. The risks have similar magnitudes for all mild GDM diagnostic classifications.

## Introduction

Gestational diabetes (GDM) is one of the most common endocrinopathies during pregnancy, affecting 4–12% of all pregnancies (1). It occurs because of metabolic maladaptation to insulin resistance, and mainly due to the hormonal changes of pregnancy (2). It is well documented that GDM is strongly associated with adverse feto-maternal and neonatal outcomes such as macrosomia, preterm birth, and small for gestational age (SGA) (3–5). Although glucose tolerance among pregnant women with GDM reverts to normal shortly after delivery, they are still potentially susceptible to type 2 diabetes mellitus (T2DM), cardiovascular disease, and obesity (6–9).

In recent decades, there have been ongoing discussions concerning the optimum diagnostic criteria for GDM across the globe. The study of Hyperglycemia and Adverse Pregnancy Outcomes (HAPO) showed that the increase of maternal glycemia was associated with the enhancement of the risk of adverse perinatal outcomes, with no obvious threshold at which risks increased (10). Therefore, it becomes more difficult to determine the optimal threshold for the diagnosis of and the treatment of GDM and accordingly, some international societies recommend more stringent criteria with lower diagnostic thresholds of glucose (11–14).

More controversies are observed when “mild” is added to GDM. It refers to the gestational hyperglycemia that does not fulfill the diagnostic criteria for GDM. With more strict criteria are used for mild GDM, the sensitivity of diagnosis is likely to be increased at the expense of the specificity, which may allow the identification of previously ignored risks, or may result in the over medicalization of healthy pregnancies.

Meanwhile, the exact definition of the milder form of GDM and its effect on adverse pregnancy outcomes are not clearly understood. Current evidence shows conflicting results about the relationship between mild GDM and adverse pregnancy outcomes (15). Although the risk of adverse pregnancy outcomes among women with mild GDM has been shown to be greater than the non-GDM population (15–21), this finding has not been confirmed (16, 22, 23). Therefore, there is a need to improve our knowledge about the accurate estimation of the risk of adverse maternal and neonatal outcomes. The aim of this systematic review and meta-analysis was to investigate the impact of mild GDM on the risk of adverse maternal and neonatal outcomes.

## Materials and Methods

This systematic review and meta-analysis was conducted based on the Preferred Reporting Items for Systematic Reviews and Meta-Analyses (PRISMA) (24). The review question was: Does untreated mild GDM increase the risk of adverse maternal and neonatal outcomes compared to non-GDM counterparts? The PICO statement was framed as follows: Patients: pregnant women with mild GDM, Intervention: none, Comparison: non-GDM pregnant women, Outcome: adverse maternal and neonatal outcomes.

The following research objectives were addressed:

° To study the pooled risk of adverse single and composite maternal outcomes among pregnant women with mild GDM compared to non-GDM counterparts, regardless of diagnostic criteria;
° To study the pooled risk of adverse single and composite neonatal outcomes among pregnant women with mild GDM compared to non-GDM counterparts, regardless of diagnostic criteria;
° To study the pooled risk of adverse single and composite maternal outcomes among pregnant women with mild GDM compared to non-GDM counterparts, with the consideration of various diagnostic criteria;
° To study the pooled risk of adverse single and composite neonatal outcomes among pregnant women with mild GDM compared to non-GDM counterparts, regardless of diagnostic criteria and with the consideration of various diagnostic criteria.

### Eligibility Criteria

They were:

(i) definition of mild GDM or gestational hyperglycemia;
(ii) specification of screening strategies and the blood sugar's threshold in the screening test;
(iii) report of one short-term single maternal and neonatal outcome of GDM;
(iv) description of the frequency or prevalence of adverse events;
(v) comparison of adverse pregnancy outcomes between the mild GDM group and the non-GDM group without receiving any treatment;
(vi) report of clear data about undergoing treatment or not.

The use of anti-diabetic treatments including physical exercise, diet therapy and/or any medication for patients with mild GDM, and the presence of glucose intolerance or diabetes in the population of studies led to exclusion. Also, reviews, commentaries, editorials, letters, conference proceedings, and case reports were excluded.

### Search Strategy

The databases of PubMed (including Medline), Web of Science, and Scopus were searched in order to retrieve empirical studies published in English language without time limitations and until May 2020. In addition, the search coverage was improved through performing a manual search in the bibliographic details of selected studies. Relevant keywords and MeSH terms were identified and used to develop search phrases using the Boolean method with AND/OR operators (Supplementary Table 1).

### Selection of Studies and Extraction of Data

The authors independently screened titles, abstracts, and full texts of retrieved studies against the inclusion and exclusion criteria. For each eligible study, the following data were extracted: name of the first author, title of journal, year of publication, research country, method, population and samples, demographic, and health-related characteristics such as age group, body mass index (BMI), strategies used for the screening of mild GDM and related values such as blood sugar tests, and the frequency and prevalence of adverse events. Errors in the data entry and extraction were prevented through performing a control check by another author on the final data used in the meta-analysis against data in original publications.

### Study Subgroups and Outcomes

To facilitate the clinical interpretation of the results of the included studies, they were classified into 3 subgroups based on the mild GDM diagnostic criteria as follows:

Group 1: screened based on oral glucose tolerance test (OGTT) with 75 g 2-h. Mild GDM diagnosis was based on only one abnormal value in OGTT-75 g;
Group 2: screened based on 1-h glucose challenge test (GCT-50 g), followed by 3-h oral glucose test (OGT-100 g). Mild GDM diagnosis was based on only abnormal values for GCT and normal values for OGTT-g;
Group 3: screened based on 1-h GCT-50 g, followed by 3-h OGT −100 g. Mild GDM diagnosis was based on only one abnormal value in OGTT 100 g.

The composite outcome of adverse maternal outcomes and the single maternal events of cesarean section, preeclampsia and pregnancy-induced hypertension were selected. Also, the composite outcome of adverse neonatal outcomes and the single neonatal events of macrosomia, large for gestational age (LGA), SGA, hypoglycemia, hyperbilirubinemia, admission to the neonatal intensive care unit (NICU), respiratory distress syndrome (RDS), shoulder dystopia, neonatal death, and preterm birth and Apgar score in 5 min less than 7 were chosen.

### Quality and Risk of Bias Assessment

Quality of the studies was critically appraised for their methodology and presentation of their results. Two reviewers who were blinded to the journal title, study author and institution, evaluated the quality of each study independently. The quality of observational studies was assessed using the modification of the Newcastle–Ottawa Quality Assessment Scale (25). Studies with scores above 6 were considered as high quality, 3–5 moderate, and below 3 low quality. The CONSORT checklist was used to appraise RCTs and studies with scores ≥70% were judged as high-quality, 40–70% moderate, 20–40% low, and <20% very low (26). The risk of bias was assessed using the ROBINS for interventional studies (27) and Cochrane Collaboration's tool for assessing risk of bias for cohort studies (28). The authors categorized the risk of bias as high risk, low risk, and some concern of risk of bias.

### Statistical Analysis

The software package STATA (version 14; STATA Inc., College Station, TX, USA) was applied to conduct statistical analysis. Heterogeneity between the studies was assessed using Cochran's Q statistic, and heterogeneity was detected with a *p*-value <0.05. The random/fixed effects models that calculated the pooled effect were used to assess heterogeneous and non-heterogeneous results. The Harbord test helped assess publication bias. In case of significant publication bias, the trim and fill method was used for adjustment. The pooled Risk Ratio (RR) and 95% CI of events in both groups were calculated using the DerSimonian and Laird, and the inverse variance methods. Meta-regression explored the association between the risk of adverse outcome of mild GDM and its diagnostic criteria as the heterogeneity source. The effect of each individual study on the overall summary estimate of meta-analysis was examined using the sensitivity analysis. The influence analysis graph indicating re-estimated meta-analysis omitting each study was drawn. The level of statistical significance was considered at *p*-value of < 0.05.

## Results

### Search and Quality Appraisal

**Figure 1:**
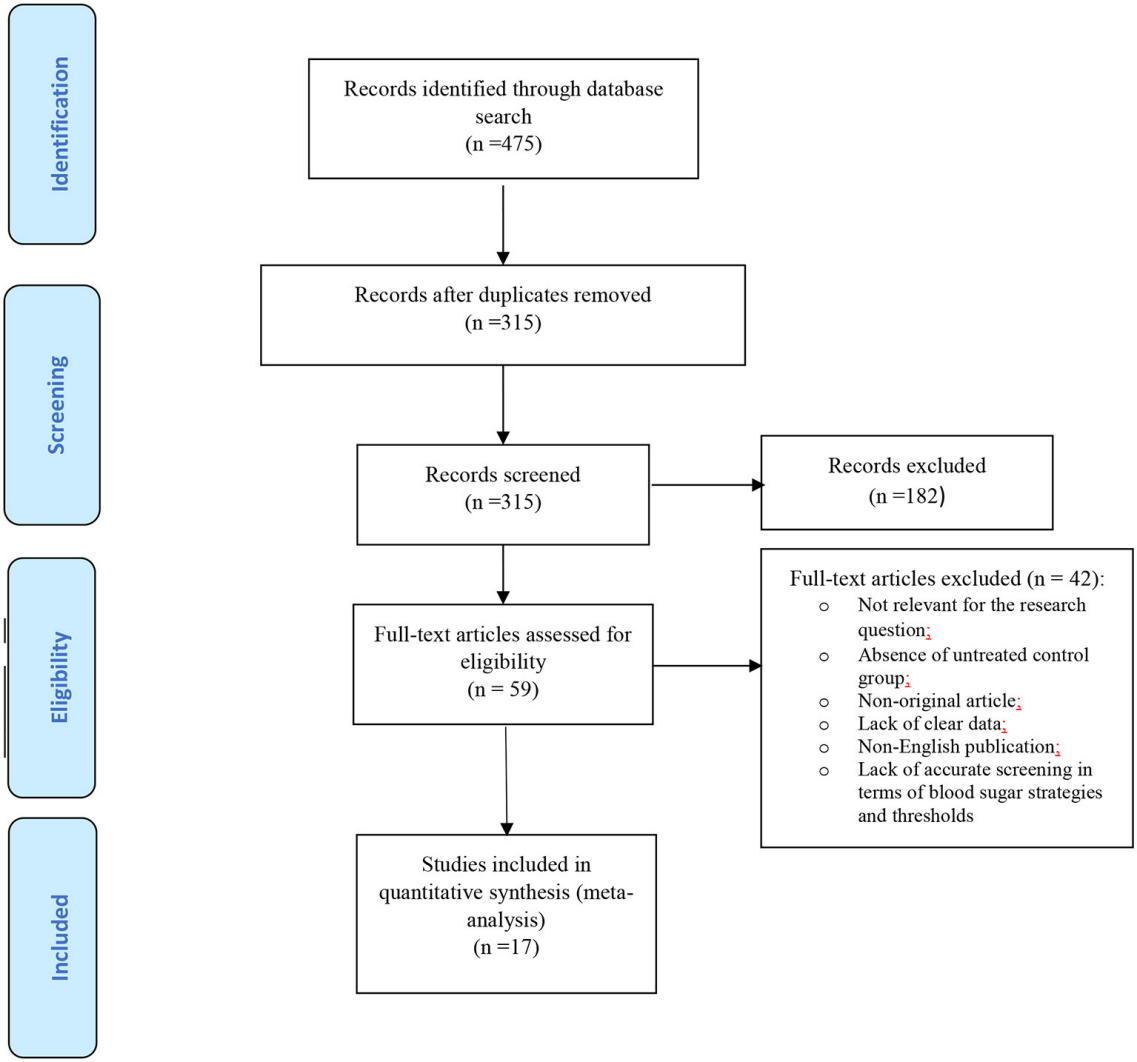
Flow diagram of the search strategy and study selection.

The flow diagram of the search strategy and study selection has been presented in Figure 1. The search strategy yielded 475 potentially relevant articles, of which 59 articles were identified suitable for further full-text appraisals. Finally, 17 studies were chosen for the meta-analysis that included data of 11,623 pregnant women with mild GDM and 53057 non-GDM counterparts. The characteristics of the included studies have been summarized in Table 1.

**Table 1:** Demographic and health-related characteristic of the studies' participants.

**References**	**Country**	**Screening test**	**Mild GDM diagnostic criteria**	**Non-GDM group characteristics**	**Mild GDM group characteristics**
Black et al. (15)	USA	OGTT-75 g-2 h	1. Either BS-1 h ≥ 180 mg/dL or BS-2 h > 153 mg/dL and FPG <92 mg/dL 2. FPG ≥ 92 mg/dL and both BS-1 h <180 mg/dL 3. BS-1 h ≥ 180 mg/dL and both BS-2 h ≥ 153 but FBS <92 mg/dL and BS-2 h <153 mg/dL 4. FBS ≥ 92 mg/dL and either BS-1 h ≥ 180 mg/dL and/or BS-2 h ≥ 153 mg/dL	*N* = 7,020, Age: 28.6 (5.9), BMI: 26.9 (5.8)	1. *N* = 391, Age: 32.1 (5.4), BMI: 28.1 (5.6) 2. *N* = 886, Age: 30.4 (5.6), BMI: 30.8 (7.1) 3. *N* = 83, Age: 32.3 (5.2), BMI: 27.5 (4.7) 4. *N* = 331, Age: 32.0 (5.1), BMI: 31.8 (7.0)
Bo et al. (16)	Italy	GCT-50-1 h g followed by OGTT-100 g-3 h	1. Only one abnormal value in OGTT 100 g: FPG > 5.3 mmol/L or BS-1 h > 10.0 mmol/L or BS-2 h > 8.6 mmol/L or BS-3 h > 7.8 mmol/L 2. GCT ≥ 7.8 mmol/L and OGTT 100 g-negative	*N* = 100, Age: 30.8 (4.2), BMI: 23.1 (4.6)	*N* = 350, Age: 31.8 (4.3), BMI: 23.5 (4.8)
Bonomo et al. (29)	Italy	GCT-50-1 h g followed by OGTT-100 g-3 h	GCT ≥ 7.8 mmol/L and OGTT 100 g-negative	*N* = 150, Age: 31.1 (4.4), BMI: 23.0 (4.1)	*N* = 150, Age: 30.7 (5.1), BMI: 23.0 (4.5)
Cakar et al. (17)	Turkey	GCT-50-1 h g followed by OGTT-100 g-3 h	GCT ≥ 7.8 mmol/L and OGTT 100 g- negative	*N* = 160, Age: 29.2 (6.1), BMI:-	*N* = 198, Age: 28.2 (5), BMI:-
Hedderson et al. (19)	USA	GCT-50-1 h g followed by OGTT-100 g-3 h	GCT ≥ 140 mg/dL and OGTT 100 g- negative	*N* = 38,515, Age: -, BMI:-	*N* = 5,352, Age: -, BMI:-
Kanai et al. (22)	Japan	GCT-50-1 h g followed by OGTT-75 g-2 h	One elevated value on FBS > 92, BS-1 h > 180 mg/dL, BS-2 h > 153 mg/dL	*N* = 135, Age: 32.6 (4.9), BMI: 20.9 (19.5–23.2)	*N* = 38, Age: 34.5 (4.8), BMI: 22.0 (20.1–23.8)
Kaymak et al. (20)	Turkey	GCT-50-1 h g followed by OGTT-100 g-3 h	1. GCT ≥ 7.8 mmol/L and OGTT 100 g- negative 2. Only one abnormal value in OGTT 100 g: FPG ≥ 5.3 mmol/L or BS-1 h ≥ 10.0 mmol/L or BS-2 h ≥8.6 mmol/L or BS-3 h ≥7.8 mmol/L	*N* = 479, Age: 25.2 (4.8), BMI:-	1. *N* = 401, Age: 27.4 (5.5), BMI:- 2. *N* = 80, Age: 29.4 (5.3), BMI:-
Landon et al. (21)	USA	GCT-50-1 h g followed by OGTT-100 g-3 h	1. GCT ≥ 135 mg/dL and OGTT 100 g- negative 2. FBS <95 mg/dL and two or more value of BS-1 h > 180 mg/dL, BS-2 h > 155 mg/dL, BS-3 h > 140 mg/dL	*N* = 437, Age: 25.1 (5.3), BMI:-	1. *N* = 931, Age: 27.4 (5.5), BMI:- 2. *N* = 473, Age: 28,9 (5.6), BMI:-
Lao et al. (30)	China	OGTT-75 g- 2 h	1. BS-2 h: 6–6.9 mmol/L 2. BS-2 h: 7–7.9 mmol/L	*N* = 304, Age: 28.6 (4.6), BMI: 21.5 (2.6)	1. *N* = 386, Age: 29.6 (4.6), BMI: 21.7 (2.7) 2. *N* = 304, Age: 30.8 (4.4), BMI: 21.8 (2.8)
Lao et al. (23)	China	OGTT-75 g- 2 h	BS-2 h: 125–142 mg/dL	*N* = 1,072, Age: 31.9, BMI: 22.8	*N* = 400, Age: 32.2, BMI: 23.3
Lee et al. (31)	Korea	GCT-50-1 h g followed by OGTT-100 g-3 h	GCT ≥ 7.8 mmol/L and OGTT 100 g- negative	*N* = 819, Age: 33.9 (3.8), BMI: 20.5 (2.4)	*N* = 476, Age: 33.2 (3.9), BMI: 21.3 (3.5)
Martínez-Cruz et al. (32)	México	OGTT-75 g-2 h	One elevated value on FBS > 92, BS-2 h > 153 mg/dL	*N* = 282, Age: 30.4 (6.5), BMI: 27.1 (4.0)	*N* = 282, Age: 29.9 (7.2), BMI: 27.3 (4.6)
Miyakoshi et al. (33)	Japan	GCT-50-1h g followed by OGTT-75 g-2 h	One elevated value on FBS > 100, BS-1 h > 180 mg/dL, BS-2 h > 150 mg/dL	*N* = 2,463, Age: 32.4 (4.3), BMI: 20.2 (2.4)	*N* = 139, Age: 33.8 (37), BMI: 20.5 (3.0)
Ostlund et al. (34)	Sweden	Random blood glucose level followed by OGTT-75 g-2 h	FBS <6.7 mmol/L and BS-2 h: 9.0–11.0 mmol/L.	*N* = 812, Age: 30.0 (5.0), BMI: 24.1 (4.0)	*N* = 213, Age: 32.5 (5.0), BMI: 27.5 (5.4)
Park et al. (35)	South korea	GCT-50-1 h g followed by OGTT-100 g-3 h	One elevated value on FBS > 95 mg/dL, BS-1 h > 180 mg/dL, BS-2 h > 155 mg/dL, BS-3 h > 140 mg/dL	*N* = 93, Age: 32.8 (3.5), BMI: 20.9 (19.6-23.7)	*N* = 38, Age: 33.6 (4.0), BMI: 22.4 (19.8-25.0)
Vambergue et al. (36)	France	GCT-50-1 h g followed by OGTT-100 g-3h	One elevated value for FBS ≥ 5.3 mmol/L, BS-1 h ≥ 10 mmol/L, BS-2 h ≥ 8.6 mmol/l, BS-3 h ≥ 7.8 mmol/L	*N* = 108, Age:-, BMI: -	*N* = 130, Age:-, BMI:-
Vambergue et al. (37)	France	GCT-50-1 h g followed by OGTT-100 g-3 h	One elevated value for FBS ≥ 5.3 mmol/L, BS-1 h ≥ 10 mmol/L, BS-2 h ≥ 8.6 mmol/L, BS-3 h ≥ 7.8 mmol/L	*N* = 108, Age: 27 (5.2), BMI: 27 (5.2)	*N* = 131, Age: 28.8 (5.8), BMI: 24.8 (4.8)

*GDM, Gestational diabetes Mellitus; BMI, Body mass index; GCT, glucose challenge test; OGTT, oral glucose challenge test; FBS, fasting blood sugar; BS, blood sugar*.

Supplementary Tables 2, 3 show the details of quality assessment performed on the included studies. The quality of fifteen studies were high (15–17, 19, 20, 22, 23, 30–37), one moderate (21), and one low quality (29), but no study had very low quality.

Fifteen studies used the cohort design (15–17, 19, 20, 22, 23, 30–37) and two others used the interventional design (21, 29). Three studies were conducted in the USA (15, 19, 21), 6 in East Asia including Japan, South Korea, and China (22, 23, 30, 31, 33, 35), 5 in Europe (16, 29, 34, 36, 37), 2 in Turkey (17, 20), and one in Mexico (32).

Thirteen studies applied the two-step screening approach using one 1-h GCT with 50 g glucose followed by 3-h OGTT with 100 glucose (16, 17, 19–22, 29, 31–33, 35, 37); among these studies, a total of 5 studies used the just elevated abnormal value of GCT (16–18, 29, 31), and others used normal GCT with one (16, 20, 22, 35–37) or two elevated abnormal values of OGTT 100 g (21) as the mild GDM diagnostic definition. As well, 4 applied the one-step screening approach using 2-h OGTT with 75 glucose (15, 23, 30, 34) and all of them used the one elevated abnormal value in those tests as mild GDM diagnostic criteria. According to those used definitions, 6 studies were classified into group 1 (15, 22, 23, 30, 32, 33), 7 into group 2 (16, 17, 19–21, 29, 31), and 7 into group 3 (16, 20, 21, 33, 35–37).

### Meta-Analysis

The overall pooled RR (95% CI) of adverse maternal and neonatal outcomes, heterogeneity, and estimation of publication bias in women with mild GDM compared to non-GDM counterparts has been shown in Table 2.

**Table 2:** Heterogeneity, estimation of publication bias, and meta-analysis for comparing the relative risk of adverse maternal and neonatal outcomes.

**Outcome**	**Sample size**		**Publication bias**	**Heterogeneity**	**Pooled overall**
			Harbord test^*^	*P*-value^*^	RR (95% CI)^*^
	**Mild GDM**	**Non-GDM**			
Composite adverse maternal outcome	25,451	160,353	0.061	**0.001**	**1.3 (1.2, 1.5)**
Cesarean section	8,223	43,465	0.344	**0.001**	**1.3 (1.2, 1.5)**
Pregnancy induced hypertension	8,819	72,398	0.287	**0.001**	**1.4 (1.1, 1.7)**
Shoulder dystocia	2,132	2,111	0.927	0.611	**2.7 (1.5, 5.1)**
Preeclampsia	6,277	42,379	0.932	0.747	**1.3 (1.1, 1.5)**
Composite adverse neonatal outcome	46,477	275,351	0.003^*^	**0.001**	1.1 (1.0, 1.2)^*^
Macrosomia	8,113	45,048	0.213	**0.001**	**1.4 (1.1, 1.7)**
LGA	11,750	74,944	0.170	**0.000**	1**.7 (1.3, 2.3)**
SGA	8,382	45,605	0.068	**0.029**	1.0 (0.7, 1.2)
Hypoglycemia	1,322	2,488	0.269	0.509	**1.6 (1.1, 2.3)**
Hyperbilirubinemia	3,001	29,729	0.029^*^	0.190	1.1 (1.0, 1.3)^*^
Neonatal death	831	1,058	0.143	0.339	1.0 (0.3, 2.9)
5 min Apgar <7	1,012	2,138	0.329	0.937	**1.6 (1.1, 2.4)**
NICU admission	922	1,414	0.631	0.972	**1.5 (1.1, 2.1)**
RDS	880	1,281	0.393	0.699	**3.2 (1.8, 5.5)**
Preterm birth	10,264	1,646	0.956	**0.001**	**1.4 (1.1, 1.7)**

*GD, Gestational diabetes; LG, Large for gestational age; SGA, Small for gestational age; NICU, Neonatal intensive care Unit; RDS, Respiratory distress syndrome*.

*Bold values indicate statistical significance*.

* *Obtained from the trim and fill method of publication bias adjustment*.

**Figure 2:**
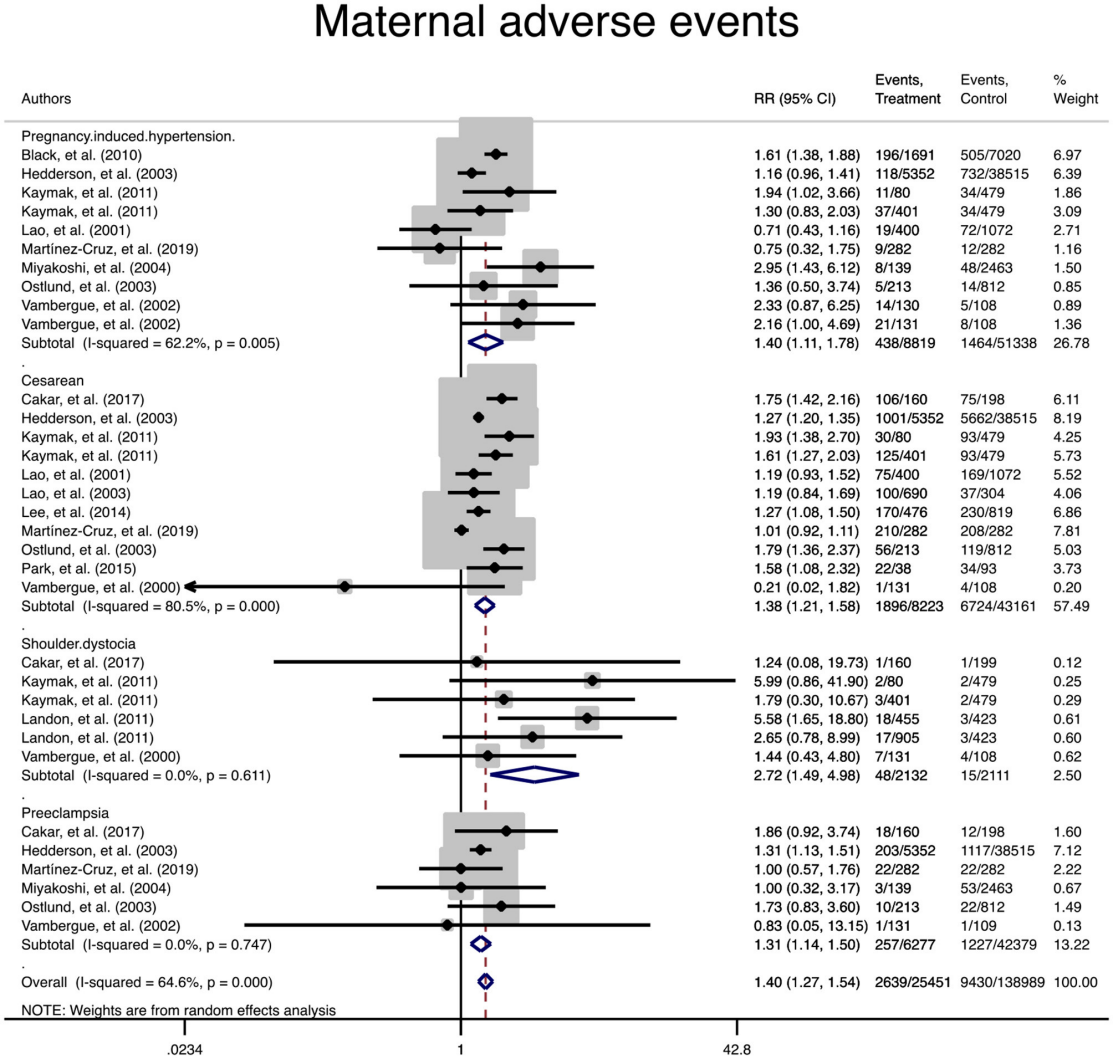
Forest plot of pooled relative risk of adverse maternal events.

In terms of adverse maternal outcomes, the women with mild GDM had a significantly higher risk of cesarean section (pooled RR: 1.3, 95% CI 1.2–1.5), pregnancy induced hypertension (pooled RR: 1.4, 95% CI 1.1–1.7), preeclampsia (pooled RR: 1.3, 95% CI 1.1–1.5), and shoulder dystocia (pooled RR: 2.7, 95% CI 1.5–5.1) in comparison with the non-GDM population, Table 2 and Figure 2.

**Figure 3:**
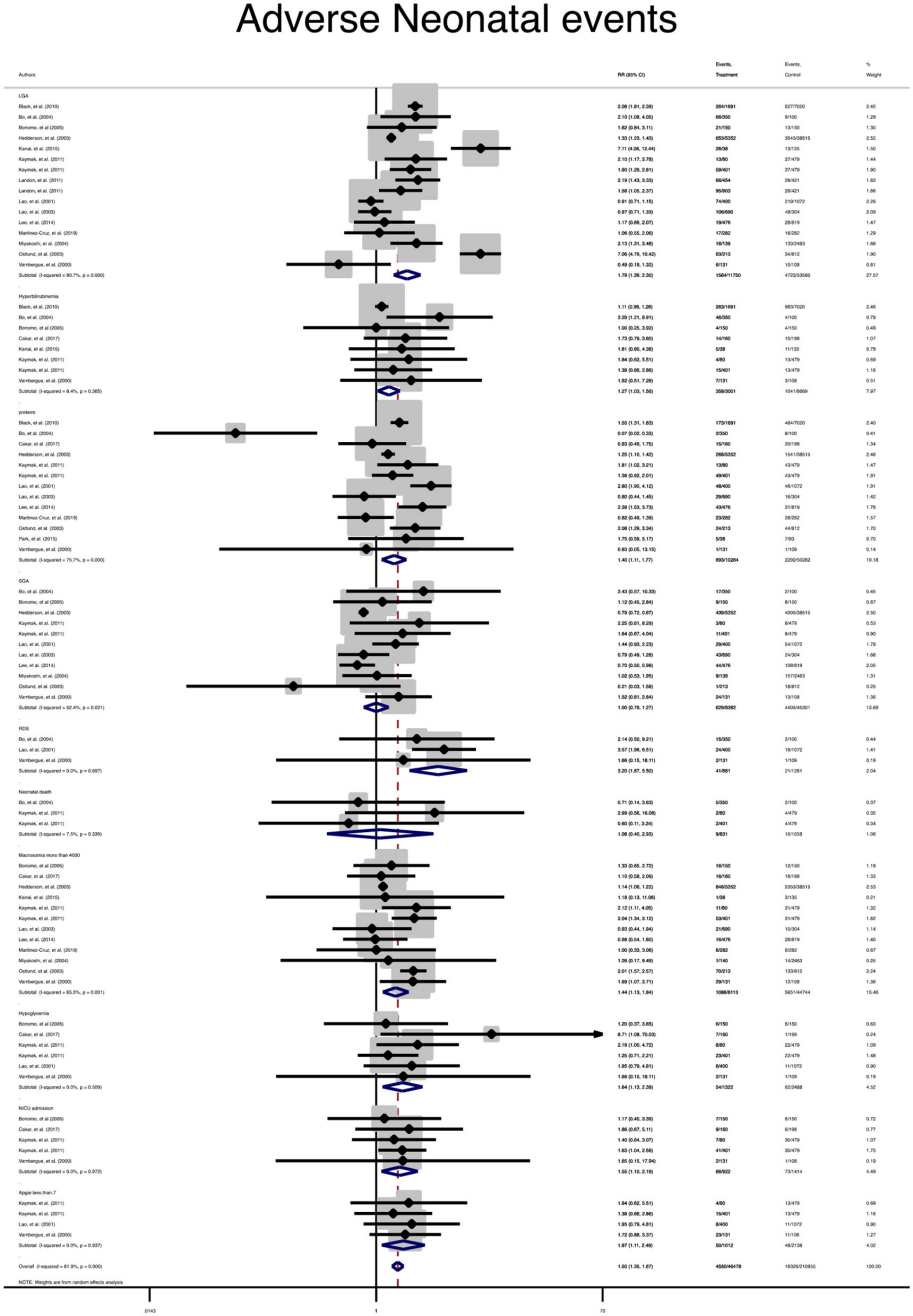
Forest plot of pooled relative risk of adverse neonatal events.

In terms of adverse neonatal outcomes, the pooled relative risk of macrosomia (pooled RR = 0.4, 95% CI: 1.1–1.7), LGA (pooled RR = 1.7, 95% CI: 1.3–2.3), hypoglycemia (pooled RR = 1.6, 95% CI: 1.1–2.3), hyperbilirubinemia (pooled RR = 1.1, 95% CI: 1–1.3), 5 min Apgar <7 (pooled RR = 1.6, 95% CI: 1.1–2.4), admission to the NICU (pooled RR = 1.5, 95% CI: 1.1–2.1), RDS (pooled RR = 3.2, 95% CI: 1.8–5.5) and preterm birth (pooled RR = 1.4, 95% CI: 1.1–1.7) significantly increased in the treated group as compared with the non-GDM group, Table 2 and Figure 3. However, the adverse events of SGA and neonatal death were not significantly different between the groups (Table 2 and Figure 3).

### Subgroup Analysis and Meta-Regression

**Figure 4:**
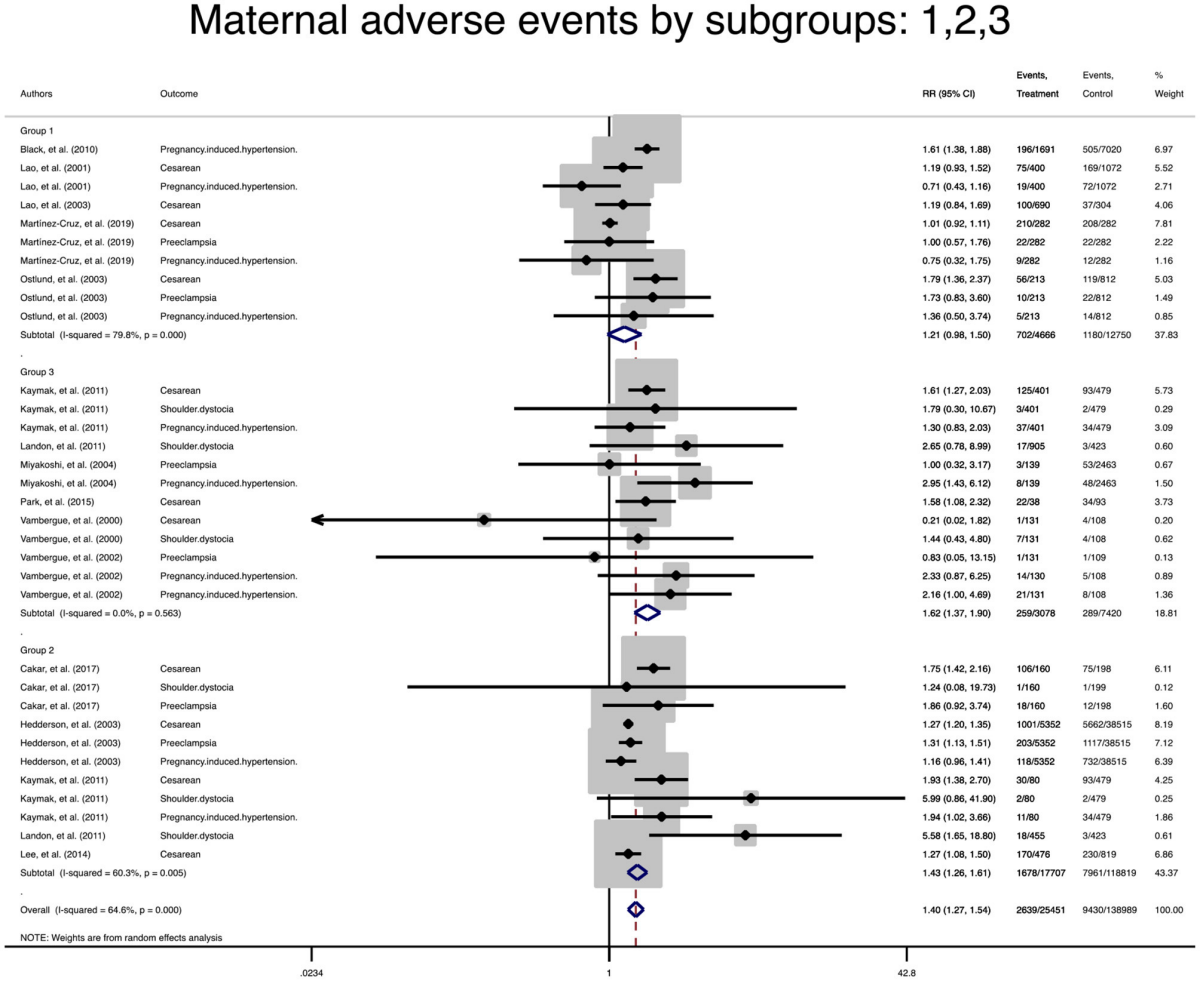
Forest plot of pooled relative risk of adverse maternal events in subgroups.

**Figure 5:**
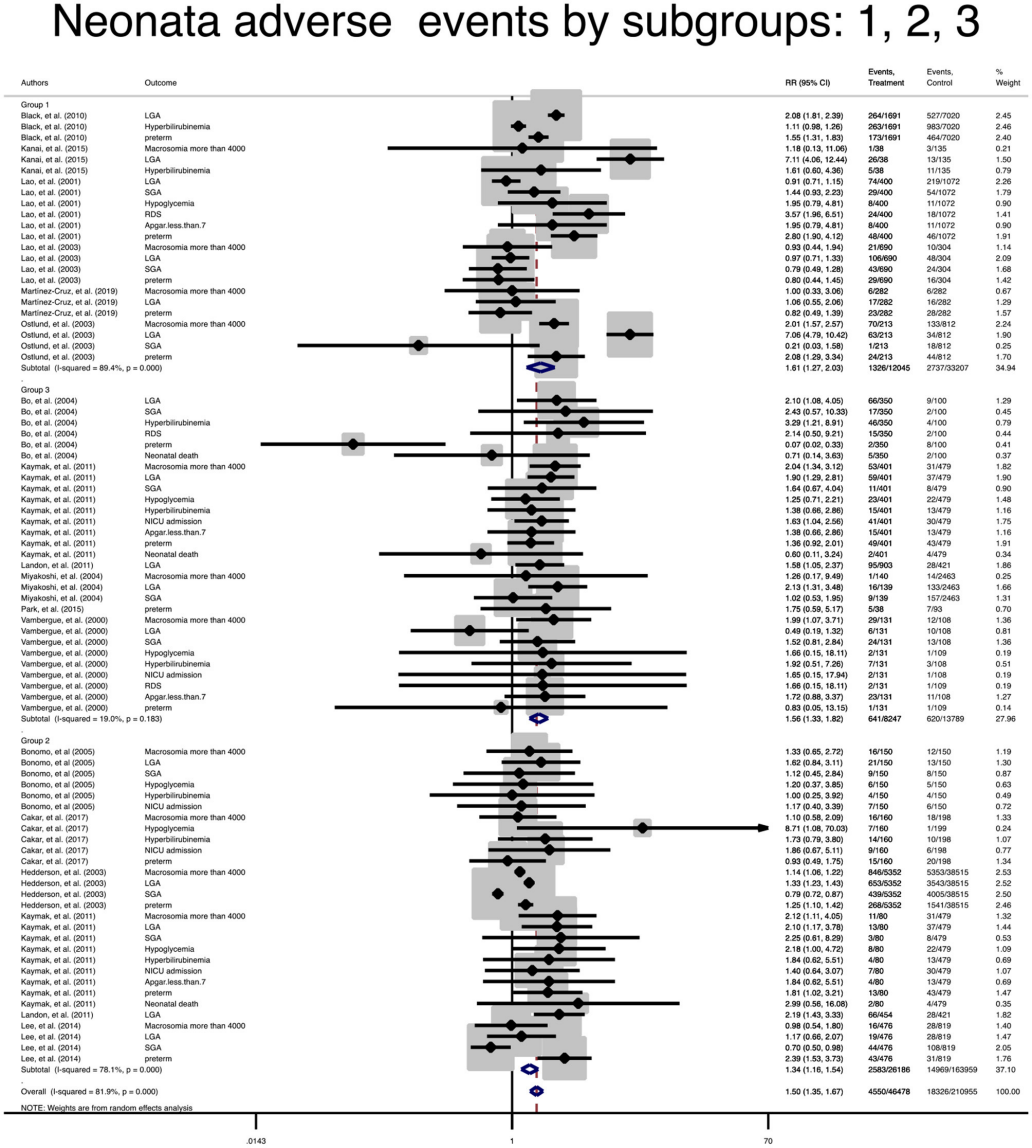
Forest plot of pooled relative risk of adverse neonatal events in subgroups.

**Figure 6:**
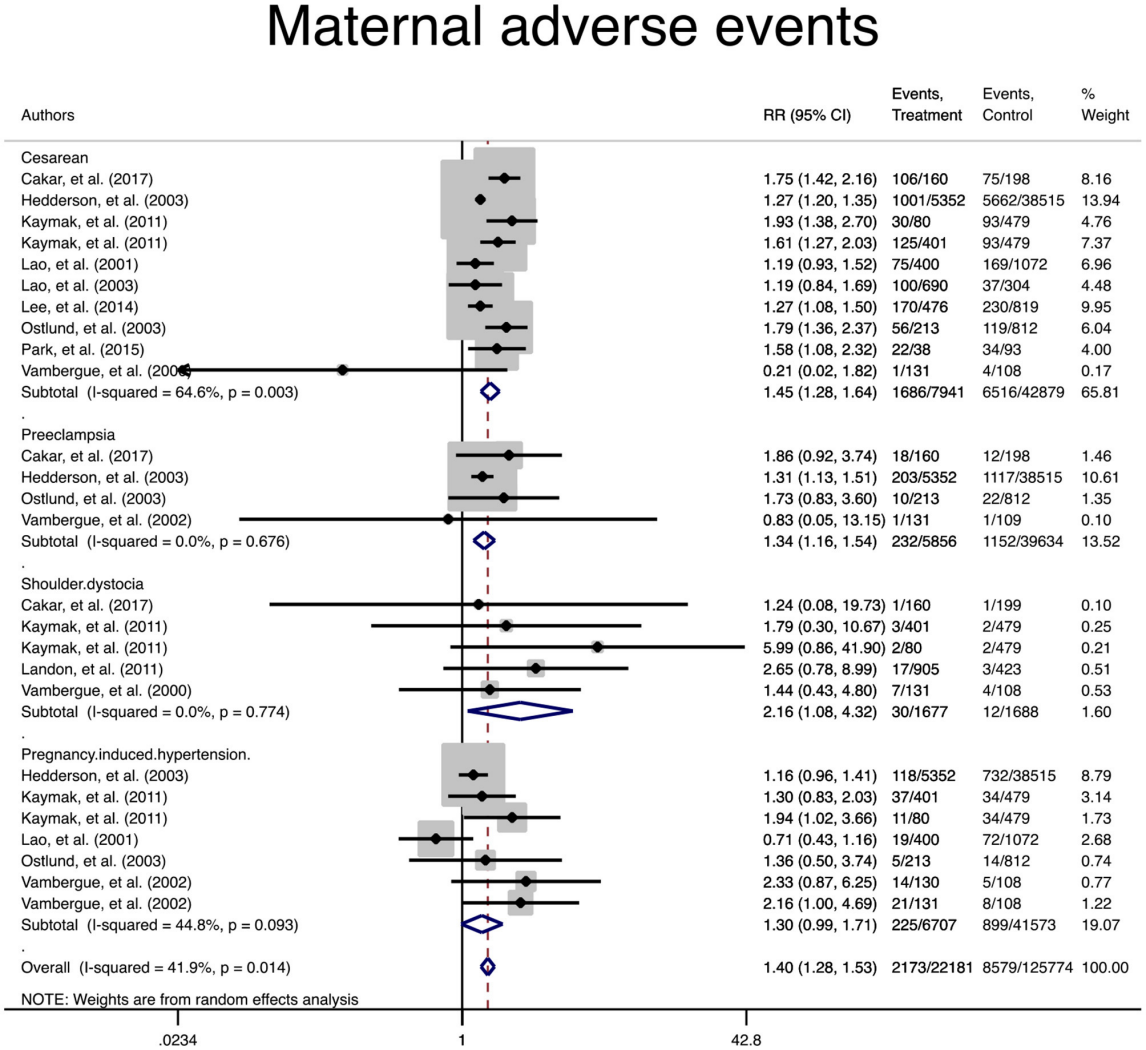
Forest plot of pooled relative risk of adverse maternal events with the exclusion of studies that fulfilled the IADPSG (HAPO) criteria.

**Figure 7:**
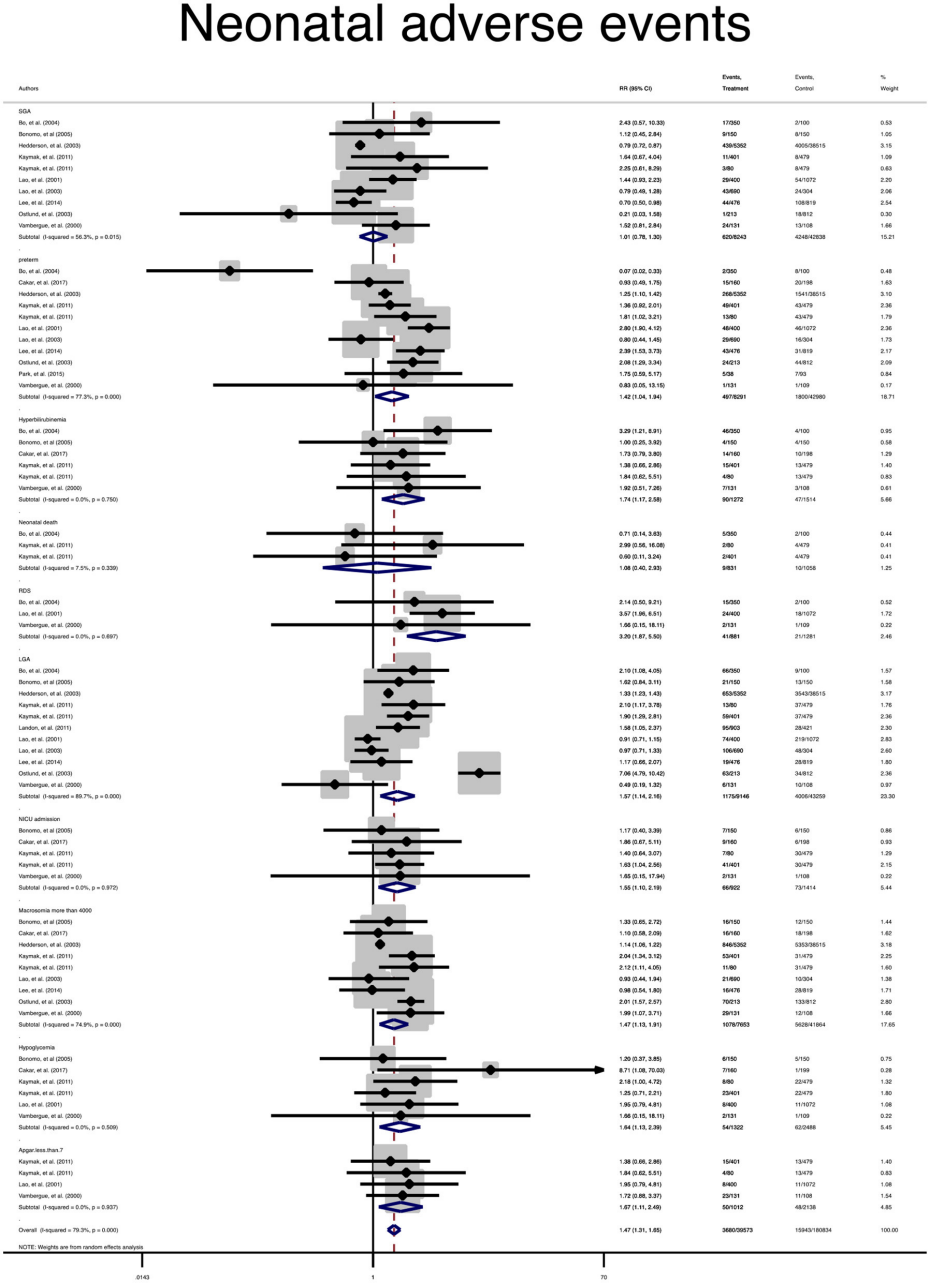
Forest plot of pooled relative risk of adverse neonatal events with the exclusion of studies that fulfilled the IADPSG (HAPO) criteria.

Due to the lack of data, subgroup analysis for single outcomes could not be performed, but the analysis of composite maternal and neonatal outcomes revealed that the risk of those adverse outcomes in women with mild GDM in all classifications were significantly higher than the non-GDM population (Table 3 and Figures 4, 5). The meta-regression showed that the magnitude of those increased risks in both composite maternal and neonatal outcomes were similar. In addition, subgroup analyses with the exclusion of studies that fulfilled IADPSG (HAPO) criteria was performed, but the results remained unchanged (Figures 6, 7).

**Table 3:** Results of heterogeneity, estimation of publication bias, and meta-analysis for comparing the relative risk of adverse maternal and neonatal outcomes based on diagnostic criteria.

**Outcome**	**Sample size**		**Publication bias**	**Heterogeneity**	**Pooled overall**	**Meta-regression**
			**Harbord test**	*P*-value^*^	RR (95% CI)^*^	***P*-value**
	**Mild GDM**	**Non-GDM**				
**Composite adverse maternal outcome**						
Sub-group 1	4,666	34,114	0.412	**0.001**	**1.2 (1.0, 1.5)**	0.840
Sub-group 2	17,707	118,819	0.186	**0.001**	**1.4 (1.2, 1.6)**	
Sub-group 3	3,078	7,420	0.411	**0.001**	**1.6 (1.3, 1.9)**	
**Composite adverse neonatal outcome**						
Sub-group 1	1,2045	97,603	0.796	**0.001**	**1.5 (1.2, 1.8)**	0.112
Sub-group 2	2,583	163,959	0.985	**0.001**	**1.3 (1.1, 1.5)**	
Sub-group 3	8,247	13,789	0.189	0.198	**1.5 (1.3, 1.8)**	

*GDM, Gestational diabetes; RR, Relative risk*.

* *Bold values indicate statistical significance*.

### Sensitivity Analysis, Publication Bias and Risk of Bias

Sensitivity analyses showed the robustness of pooled RR indicating no major impact of any single study on pooled RR in both maternal and neonatal adverse outcomes (Supplementary Figures 1, 2).

According to the Harbord test, there was no publication bias for most analyses in the meta-analysis. However, a significant publication bias was found, in the meta-analyses of composite adverse neonatal outcome and hyperbilirubinemia, which was corrected by the trim and fill method of adjustment (Table 2). A low risk of bias was observed in the appraised domains (Supplementary Figures 3, 4). Cohort studies had a low risk of bias for the selection of exposed and non-exposed cohorts, assessment of exposure, presence of the outcome of interest at the start of the study, outcome assessment, and adequacy of follow up of cohorts. However, around 7% of them had a probable low risk of bias in controlling prognostic variables and one third of them had a high risk of bias in the assessment of the presence or absence of prognostic factors and controlling prognostic variables. Two interventional studies had a low risk of bias in the measurement of outcomes and selection of the reported results and had some concern regarding bias in the randomization process, intervention, and missing outcome data.

## Discussion

This meta-analysis indicated that although mild GDM in spite of not fulfilling the diagnostic criteria for GDM did not increase the risk of sever adverse neonatal outcomes of SGA and neonatal mortality, it increased the risks of most adverse maternal and neonatal outcomes including cesarean section, shoulder dystocia, macrosomia, LGA, preeclampsia, pregnancy induced hypertension, preterm birth, hypoglycemia, hyperbilirubinemia, low Apgar score, RDS, and admission to the NICU compared to subjects with completely normal glucose tolerance. Moreover, subgroup analysis based on the definition demonstrated that all mild GDM criteria used by available studies similarly increased the risk of composite maternal and neonatal outcomes.

Gestational hyperglycemia is usually the result of β-cell dysfunction, caused partly by hormones from the placenta and partly by other obesity and pregnancy related factors that are not fully understood on the background of chronic insulin resistance during pregnancy. Altered carbohydrate metabolism may cause arteriosclerosis and dysfunction in the glomerular filtration leading to adverse maternal outcomes during pregnancy (38). Moreover, it is demonstrated that maternal hyperglycemia is readily transported across placenta to fetus and stimulates the fetus's endogenous production of insulin and insulin-like growth factor 1 (IGF-1) (2, 39). Together, these can cause fetal overgrowth, often resulting in macrosomia at birth. As well, excess fetal insulin production can cause hypoglycemia, which can contribute to brain injury if not properly managed (40). Moreover, fetal hyperinsulinemia has been suggested to be associated with delayed pulmonary maturation, which is also the risk factor for neonatal respiratory morbidity (41).

Although it has long been recognized that women with GDM are at the increased risk of adverse maternal and fetal outcomes if optimal care is not provided, the relationship of milder form of GDM to various perinatal risks has been less documented. As noted by the HAPO study (10), GDM is a wide range of maternal hyperglycemia, in which blood glucose levels stay along a continuum and is correlated with a wide spectrum of metabolic abnormalities and conferring the varying degrees of pregnancy-related risk (42). In this respect, there were no obvious thresholds at which the risk for any of perinatal outcomes increased in a more intense manner, instead of rising along a continuum. Consistent with the findings of the HAPO study (10), our meta-analysis confirm that the ‘borderline' situations of hyperglycemia can alter glucose metabolism in pregnancy, and subsequently increase the risk of many important adverse pregnancy outcomes compared to the non-GDM population.

In agreement with our finding, a recently published meta-analysis of 10 interventional studies reported that the standard treatment of GDM through diet therapy and insulin improved adverse pregnancy outcomes in women with the milder form of GDM (43). In this study, the risk of adverse pregnancy outcome in a total of 3,317 pregnant women with borderline hyperglycemia who received the standard GDM treatment was compared with 4,407 untreated counterparts. Therefore, treatment reduced the risk of macrosomia, LGA and shoulder dystocia without enhancing the risk of SGA in these women (43).

The lack of a standard definition for mild GDM during pregnancy causes that different studies on this topic produce various definitions. Therefore, the studies' samples consist of women with various levels of glucose intolerance. Moreover, it is suggested that the risks of different adverse pregnancy outcomes vary depending on which single or combined OGTT thresholds are equaled or exceeded (42). It should be noted that all those definitions did not fulfill the GDM criteria. Moreover, to determine whether those definitions could increase the risk of adverse outcomes, mild GDM definition was classified. However, the subgroup analysis revealed that the risk of composite adverse outcomes in women with mild GDM in all definitions were significantly higher than the non-GDM population and importantly the magnitude of those increased risks remained similar. It should be noted that the lack of data hindered the analysis of single outcomes; therefore, the results of composite outcomes must be interpreted with caution, which raises concern that composite outcome may not reflect the mild disease and may confer the higher risk compared to adverse single outcome.

In addition, mild GDM did not increase the risk of sever adverse neonatal outcomes including neonatal death and SGA. It is believed that these outcomes often are associated with severe glucose intolerance than that included to this review. The participants of the present meta-analysis had a normal level of fasting maternal glucose. It has been reported that the threshold of an enhanced risk of neonatal hypoglycemia is not observed until the fasting maternal glucose level exceeds 100 mg/dL (5.6 mmol/L) (10, 15, 44).

The study limitations should be considered during the interpretation of findings. A lack of unique definition for mild GDM and adverse pregnancy outcomes influenced the data analysis. The sample size of the studies was low. Also, data was collected in developed counties, which should not be extrapolated to women living in developing countries with different lifestyles, ethnicities, and access to healthcare facilities. Moreover subgroup-analysis based on fasting maternal glucose results known as the adverse pregnant outcomes' indicator (15) were not carried out, because of lack of data. In addition, risk factors for GDM including overweight and obesity, advanced maternal age, and a family history or any form of diabetes were not evaluated in our study, due to insufficient data in the original studies. The short-term adverse outcomes of mild GDM were assessed, but longer-term outcomes should be investigated in future studies. Nonetheless, not enough power for reporting statistically significant findings for other pregnancy outcomes could be achieved.

## Conclusion

The findings of our study support that the borderline situations of gestational hyperglycemia, lower than diagnostic criteria for GDM, can increase most adverse maternal and neonatal outcomes. However, it does increase the risk of severe neonatal outcomes of SGA and neonatal mortality. These findings can give some clue to healthcare professionals for redefining criteria for the diagnosis of GDM and to include those women with milder form of disease. Well-defined studies with larger sample sizes are needed to confirm our review results.

## Data Availability Statement

The original contributions presented in the study are included in the article/Supplementary Material, further inquiries can be directed to the corresponding author/s.

## Author Contributions

SB-G: conceptualization and methodology. SB-G, MV, and IG: writing-original draft preparation, editing, and revising it critically for important intellectual content. RB and MP quality appraisal, data analysis, and interpretation. All authors read and approved the final manuscript to be published.

## Conflict of Interest

The authors declare that the research was conducted in the absence of any commercial or financial relationships that could be construed as a potential conflict of interest.
