# Supplementary material for: Mild Gestational Diabetes and Adverse Pregnancy Outcome: A Systemic Review and Meta-Analysis
Source: Front Med (Lausanne). 2021 Jul 5;8:699412. doi: 10.3389/fmed.2021.699412 (PMC8286997; doi:10.3389/fmed.2021.699412)

**Supplementary file**

Supplementary Table 1. Keywords and search phrases used in the review process

| Keywords and search phrases |
| --- |
| (adverse pregnancy outcomes OR pregnancy outcomes OR pregnancy complications OR gestational age OR macrosomia OR large for gestational age OR LGA OR small for gestational age OR SGA OR neonatal hypoglycemia OR hypoglycemia OR Hyperbilirubinemia OR icterus OR elevated C- peptide OR c-peptide OR C peptide OR NICU OR NICU admission OR respiratory distress syndrome OR RDS OR Apgar OR preterm birth OR preterm labor OR still birth OR IUFD OR intrauterine fetal death OR mortality OR IUGR OR intrauterine growth restriction OR polyhydramnios OR oligohydramnios OR preeclampsia OR pregnancy induced hypertension OR gestational hypertension OR PIH OR hemorrhage OR postpartum hemorrhage OR PPH OR placenta abruption OR placenta previa OR antepartum hemorrhage OR maternal weight gain OR pregnancy weight gain OR gestational weight gain OR birth weight OR induction of labor OR labor induction OR induced labor OR instrumental delivery OR operative delivery OR cesarean sections OR C-section OR abdominal deliveries OR birth trauma OR shoulder dystocia) AND (mild gestational diabetes OR mild GDM OR mild gestational hyperglycemia OR mild maternal hyperglycemia OR mild glucose intolerance in pregnancy OR mild gestational glucose intolerance OR mild gestational carbohydrate intolerance OR mild carbohydrate intolerance in pregnancy OR mild gestational impaired glucose tolerance OR mild impaired glucose tolerance in pregnancy). |

Supplementary Table 2. Quality assessment of included studies using the Newcastle–Ottawa Quality Assessment Scale for cohort studies.

|  | SELECTION | | | | COMPARABILITY | OUTCOME | | | Total scores |
| --- | --- | --- | --- | --- | --- | --- | --- | --- | --- |
| Author | Representativeness of the exposed cohort | Selection of the non-exposed cohort | Ascertainment of exposure | Demonstration that outcome of interest was not present at the start of study | A: Study controls for age and/or Sex  B: Study controls for other confounders | A: Independent blind assessment  B: Record linkage | follow-up long enough for outcomes | Adequacy of follow up of cohorts |  |
| Black et al. (2010) 15 | * | * | * | * | * | * | * | * | 8 |
| Bo et al. (2004) 16 | * | * | * | * | ** | * | * | * | 9 |
| Cakar et al. (2017) 17 | * | * | * | * | - | * | * | * | 7 |
| Hedderson, et al. (2003) 19 | * | * | * | * | ** | * | * | * | 9 |
| Kanai et al. (2015) 22 | * | * | * | * | ** | * | * | * | 9 |
| Kaymak et al. (2011) 20 | * | * | * | * | ** | * | * | * | 9 |
| Lao et al. (2001) 23 | * | * | * | * | * | * | * | * | 8 |
| Lao et al. (2003) 29 | * | * | * | * | - | * | * | * | 7 |
| Lee et al. (2014) 30 | * | * | * | * | ** | * | * | * | 9 |
| Martínez-Cruz et al. (2019) 31 | * | * | * | * | - | * | * | * | 8 |
| Miyakoshi et al. (2004) 32 | * | * | * | * | - | * | * | * | 7 |
| Ostlund et al. (2003) 33 | * | * | * | * | ** | * | * | * | 9 |
| Park et al. (2015) 34 | * | * | * | * | ** | * | * | * | 9 |
| Vambergue et al. (2000) 36 | * | * | * | * | ** | * | * | * | 9 |
| Vambergue et al. (2002) 35 | * | * | * | * | - | * | * | * | 7 |

Supplementary Table 3**:** Quality assessment of included studies using the Consort Assessment Scale for interventional studies

| Author | Results | | | | | | | | | | Total | Quality |
| --- | --- | --- | --- | --- | --- | --- | --- | --- | --- | --- | --- | --- |
|  | Participant flow (a diagram is strongly recommended) | | Recruitment | | Baseline data | Numbers analyzed | Outcomes and estimation | | Ancillary analyses | Harms |  |  |
|  | A | b | a | b |  |  | a | b |  |  |  |  |
| Bonomo et al. (2005) 37 | - | - | - | - | + | + | + | + | - | - | 10 | Low |
| Landon et al. (2011) 21 | - | - | - | - | + | + | + | + | - | - | 12 | Moderate |

| Author | Methods | | | | | | | | | | | | | | | | |
| --- | --- | --- | --- | --- | --- | --- | --- | --- | --- | --- | --- | --- | --- | --- | --- | --- | --- |
|  | Trial design | | Participants | | Interventions | Outcomes | | Sample size | | Randomization | | Allocation concealment mechanism | Implementation | Blinding | | Statistical methods | |
|  | a | B | a | b |  | a | b | A | b | a | b |  |  | a | b | a | b |
| Bonomo et al. (2005) 37 | - | - | + | + | + | + | - | - | - | - | - | - | - | - | - | + | + |
| Landon et al. (2011) 21 | + | - | + | + | + | + | - | + | - | - | - | - | - | - | - | + | + |

Supplementary Figure 1. Sensitivity analysis for adverse maternal outcomes

Supplementary Figure 2. Sensitivity analysis for adverse neonatal outcomes

**Risk of bias**

Supplementary Figure 3. Risk of bias in cohort studies.

**A.**

| Author, date | Bias in the selection of exposed and non‐exposed cohorts | Bias in the assessment of exposure | Bias in presence of the outcome of interest at start of study | Bias in the control of prognostic variables (with matching or adjusting) | Bias in the assessment of the presence or absence of prognostic factors | Bias in in the assessment of outcome | Bias in adequacy about follow up of cohorts |
| --- | --- | --- | --- | --- | --- | --- | --- |
| Black et al. (2010) 15 |  |  |  |  |  |  |  |
| Bo et al. (2004) 16 |  |  |  |  |  |  |  |
| Cakar et al. (2017) 17 |  |  |  |  |  |  |  |
| Hedderson et al. (2003) 19 |  |  |  |  |  |  |  |
| Kanai et al. (2015) 22 |  |  |  |  |  |  |  |
| Kaymak et al. (2011) 20 |  |  |  |  |  |  |  |
| Lao et al. (2001) 23 |  |  |  |  |  |  |  |
| Lao et al. (2003) 29 |  |  |  |  |  |  |  |
| Lee et al. (2014) 30 |  |  |  |  |  |  |  |
| Martínez-Cruz et al. (2019) 31 |  |  |  |  |  |  |  |
| Miyakoshi et al. (2004) 32 |  |  |  |  |  |  |  |
| Ostlund et al. (2003) 33 |  |  |  |  |  |  |  |
| Park et al. (2015) 34 |  |  |  |  |  |  |  |
| Vambergue et al. (2000) 36 |  |  |  |  |  |  |  |
| Vambergue et al. (2002) 35 |  |  |  |  |  |  |  |
| Definitely No (low risk of bias) probably no  Definitely yes (high risk of bias) probably Yes | | | | | | | |

B.

Supplementary Figure 4. Risk of bias in interventional studies using ROB 2 tool

**A.**

**
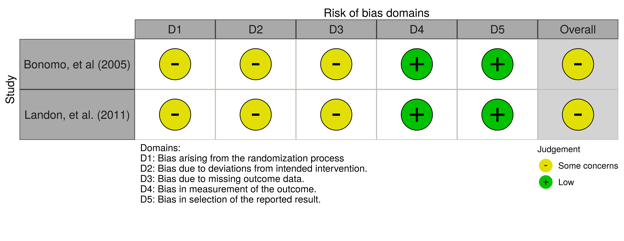
**

**B.**


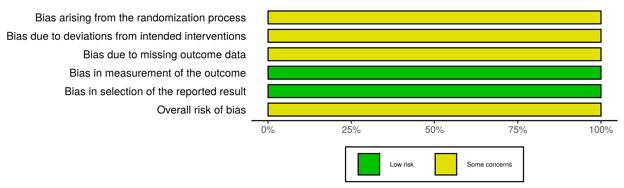

Supplement: Supplementary file 1 [file Table_1.docx]
